# Mouse APOBEC3 Restriction of Retroviruses

**DOI:** 10.3390/v12111217

**Published:** 2020-10-27

**Authors:** Karen Salas-Briceno, Wenming Zhao, Susan R. Ross

**Affiliations:** Department of Microbiology and Immunology, University of Illinois at Chicago College of Medicine, 835 S. Wolcott Avenue, Chicago, IL 60612, USA; ksalas56@uic.edu (K.S.-B.); wmzhao@uic.edu (W.Z.)

**Keywords:** APOBEC3, restriction factor, cytidine deaminase, murine leukemia virus, mouse mammary tumor virus

## Abstract

Apolipoprotein B mRNA editing enzyme, catalytic peptide 3 (APOBEC3) proteins are critical host proteins that counteract and prevent the replication of retroviruses. Unlike the genome of humans and other species, the mouse genome encodes a single *Apobec3* gene, which has undergone positive selection, as reflected by the allelic variants found in different inbred mouse strains. This positive selection was likely due to infection by various mouse retroviruses, which have persisted in their hosts for millions of years. While mouse retroviruses are inhibited by APOBEC3, they nonetheless still remain infectious, likely due to the actions of different viral proteins that counteract this host factor. The study of viruses in their natural hosts provides important insight into their co-evolution.

## 1. Introduction

Proteins belonging to the apolipoprotein B mRNA editing enzyme, catalytic peptide 3 (APOBEC3)/activation-induced deaminase (AID) are DNA cytosine deaminases with roles in innate and adaptive immunity. APOBEC3 proteins are critical restriction factors of retroviruses and retrotransposable elements. Unlike the human genome, which encodes seven different *APOBEC3* genes, only one *Apobec3* gene is found in mice (reviewed in [1]), making it relatively straight forward to delete the gene in murine embryonic stem cells. Thus, the in vivo importance of APOBEC3 proteins in controlling retrovirus infection was demonstrated through the use of knockout mice with targeted deletion of the *Apobec3* gene. *Apobec3* (A3) knockout mice were found to be more susceptible to infection by their natural pathogens, the betaretrovirus mouse mammary tumor virus (MMTV) [2], and several different strains of murine leukemia virus (MLV) gammaretroviruses [3,4,5,6,7,8]. This has led to fundamental insights into how APOBEC3 proteins function in the context of the whole organism. Here we review what has been learned from the study of mouse APOBEC3 and its inhibition of naturally infectious retroviruses in mice.

## 2. Mechanism of Action of Mouse APOBEC3

**Figure 1:**
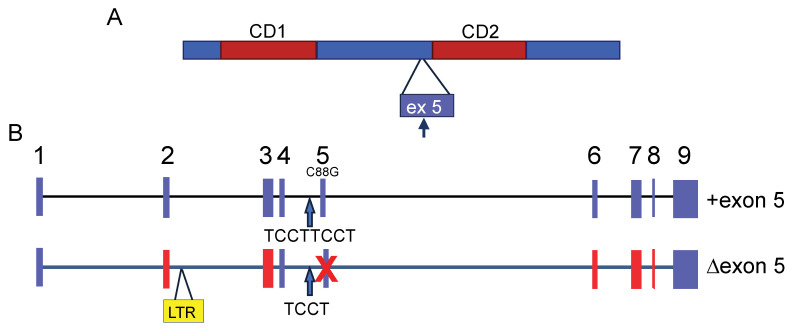
Mouse APOBEC3. (**A**) Diagram of mAPOBEC3 protein. CD1 encodes the deaminase activity and CD2 is required for packaging into virions. Exon 5 (ex5) is found in proteins made in certain inbred mouse strains. The black arrow points to the possible viral protease (PR) cleavage site in exon 5. (**B**) Diagram of the intron/exon structure of the two common m*Apobec3* alleles. Red exons denote polymorphic coding regions. Blue arrows point to the polymorphism at the end of intron 4 that likely influences retention (+exon 5) or skipping (Δexon 5) of the 5th exon. See text for other details. Abbreviations: CD, cytidine deaminase domain; LTR, long terminal repeat.

Mouse APOBEC3 (mAPOBEC3) has two cytidine deamination (CD) domains indispensable for nucleic acid binding and enzymatic activity. Each CD domain contains a conserved zinc-coordinating motif. mAPOBEC3 deaminase activity is exerted by the N-terminal domain (CD1), while the C-terminal domain (CD2) is essential for its encapsidation [9] (Figure 1A). This is in contrast to the human APOBEC3 proteins with two CD domains; the CD1 of APOBEC3G and 3F, for example, functions as the encapsidation domain, and CD2 encodes the cytidine deaminase activity [10,11]. mAPOBEC3, as with other APOBEC3s, is packaged in budding virions via interaction with both nucleocapsid (NC) and RNA and is thus transported into target cells during infection [2,9,10,12,13].

APOBEC3 proteins bind to nascent minus-strand retroviral cDNA during reverse transcription and deaminate cytosines, thereby generating uracils, which results in high levels of G-to-A mutations in the plus (coding) strand of viral DNA. These mutations generate missense and stop codons and lead to the generation of defective or truncated viral proteins, thereby producing non-infectious virions (reviewed in [14]). Like other family members, mAPOBEC3 has deaminase activity [12] and in transfection studies has been shown to restrict HIV-1 infection as strongly as APOBEC3G, causing extensive deamination of the HIV-1 genome [15,16]. In contrast, mAPOBEC3 largely restricts murine retrovirus replication through cytosine deamination-independent mechanisms, likely by binding reverse transcriptase (RT) and blocking reverse transcription [7,12,17,18]. In fact, it has been demonstrated in vitro, as well as in vivo, that mAPOBEC3 inhibits infection of exogenous murine gammaretroviruses, such as Friend (FMLV) and Moloney MLV (MMLV), and betaretroviruses, like MMTV, without extensively mutating the viral genome [3,12,19]. The lack of cytidine deamination by mAPOBEC3 is not the result of an inherent resistance of mouse retroviruses to APOBEC3-mediated deamination, since in MMTV- and MLV-infected mice expressing a human APOBEC3G transgene, both viral genomes were extensively deaminated [19,20]. A recent study also suggested that incorporation of APOBEC3 into MLV virions resulted in increased RT errors during reverse transcription, although the mechanism by which this loss of fidelity occurs was not shown [7].

Some mouse retroviruses are partially susceptible to mAPOBEC3-mediated deamination. In particular, mAPOBEC3 blocks the natural transmission of AKV, an endogenous ecotropic MLV, and both in vivo and in vitro, low-level G-to-A mutation is found; it is likely that for this virus, mAPOBEC3 limits infection by both cytidine deaminase-dependent and -independent mechanisms [6,21]. Similarly, mAPOBEC3 can deaminate MMTV, albeit at levels so low as to likely not inhibit infection [12].

mAPOBEC3 expressed in target cells also inhibits infection by incoming retroviruses [5,22,23]. This form of mAPOBEC3-mediated inhibition of MLV infection also does not require cytidine deaminase activity, since transgenic mice expressing mAPOBEC3 with mutation of both CD1 and CD2, which prevents both packaging and deamination, still inhibited MLV and MMTV infection in vivo, although to a lesser extent than the wild type endogenous protein [17].

Another cytosine deamination-independent mechanism described more recently involves the viral protease (PR). Retroviral capsid proteins are produced by a virus-encoded protease that cleaves the gag-pol polyprotein precursor. mAPOBEC3 directly binds MLV Pr180gag-pol precursor polyprotein and perturbs its autocatalytic cleavage [24]. This function of mAPOBEC3 is mainly carried out by the C-terminal half containing the CD2 interaction domain, and causes decreased production of mature viral protease (PR), inhibiting Pr65gag processing in MLV virions. This cytosine deamination-independent mechanism may happen even earlier than the block in reverse transcription, since polyprotein cleavage occurs in shed virions prior to target cell infection.

## 3. *Apobec3* Polymorphisms Affect Antiviral Activity

The selective pressure placed on the m*Apobec3* gene has resulted in polymorphisms, and these polymorphisms have been linked to susceptibility to infection by murine retroviruses [4,25,26]. Different inbred strains of mice have long been known to be resistant (C57BL) or susceptible (BALB/c, C3H, 129) to Friend virus complex (FV) infection [27]. FV is composed of the Friend murine leukemia (FMLV) helper virus and polycythemia-inducing spleen focus-forming virus; the latter virus is pathogenic and causes erythroleukemia. In 1978, the recovery from Friend Virus 3 (*Rfv3*) gene was first identified as a resistance trait in certain inbred mouse strains [28]. It was characterized by the persistence of viremia that occurred after FV infection in prototypic FV-susceptible A/WySn mice compared to resistant B10.A/SgSn mice [29]. The *Rfv3* gene was required for both recovery from viremia and resistance to leukemia induced by FV [28]. Mice with at least one dominant *Rfv3^r^* allele produce FV-specific neutralizing antibodies and recover from infection [30]. Later, the *Rfv3* gene was mapped to a narrow segment on mouse chromosome 15, where the *Apobec3* gene is localized, suggesting that polymorphisms in this gene could constitute the physiological resistance factor to FV infection [4,31,32]. Inbred mouse strains also differ in their susceptibility to MMTV infection, and *Apobec3* polymorphisms likely play a role in the genetics of susceptibility or resistance [12,26].

There are two major *Apobec3* allelic variants in common inbred mouse strains that differ in their splicing pattern, expression levels, and coding sequence. The major transcript encoded by the *Apobec3* allele expressed in FV-resistant C57BL/6 mice lacks exon 5 (Δ5), and the product of this allele is a 49-kDa protein that highly restricts FV replication both in vitro and in vivo (Figure 1B). In contrast, FV-susceptible mice, such as BALB/c, express “full-length” mAPOBEC3 (+5), which includes exon 5, and only express very low levels of the shorter isoform [3,26,33] (Figure 1B). The inclusion of exon 5 in +5 mAPOBEC3 transcripts is likely caused by an additional TCCT repeat at the end of the 4th intron as well as a polymorphic C-to-G change in exon 5 (C88G) [26,33] (Figure 1B). The Δ5 variant RNA is expressed at much higher levels than the +5 variant [12,26,33]. This is due to the insertion of a 531 bp sequence that is 96.6% identical to the long terminal repeat (LTR) of xenotropic mouse gammaretrovirus between exons 2 and 3; this provides a transcriptional enhancer associated with elevated Δ5 mAPOBEC3 transcripts in spleens of laboratory and wild-derived mice [34] (Figure 1B). Since the acquisition of the LTR is associated with resistance to retrovirus infection, this suggests that selection by these or similar viruses played a role in its retention [34].

The loss of exon 5 and the level of expression of mAPOBEC3 in hematopoietic tissues represent only part of the effect of the polymorphic differences. mAPOBEC3 exon 5 also influences protein synthesis at a post-transcriptional level. The presence of exon 5 reduces mAPOBEC3 protein levels for both allelic variants, modulating translation efficiency rather than protein degradation. The m*Apobec3* genes of virus-resistant and -susceptible mice also differ in the protein sequence outside of exon 5 [34] (Figure 1B). Phylogenetic analysis showed that most of these mAPOBEC3 polymorphic sites have been under positive selection throughout *Mus* evolution. mAPOBEC3 shows strong positive selection marked by an increase in replacement versus synonymous substitutions. Six of the ten codons that have evolved under strongest positive selection are in two clusters in the N-terminal catalytically active CD1 domain. In addition, five of these six codons specify different amino acids in MLV- and MMTV-resistant and susceptible mouse strains, and mutational analysis suggests these residues contribute to viral resistance of mAPOBEC3 [3,34].

Many MMTV-susceptible and -resistant mouse strains also express the 5+ and Δ5 mAPOBEC3s, respectively, although not all MMTV-resistant strains contain the Δ5 allele, likely due to other genetic differences [12]. Both the +5 and Δ5 proteins packaged in MMTV virions deaminated an artificial substrate with similar efficiency and both caused low-level deamination of MMTV reverse transcripts in endogenous reverse transcription assays carried out with purified virions [12]. However, the packaged Δ5 protein was more effective at blocking MMTV reverse transcription than was the +5 protein in endogenous reverse transcription assays. This seemed to be a function of the level of APOBEC3 protein rather than of the other polymorphic differences [12].

## 4. Mouse *Apobec3* Is an Interferon-Inducible Gene

Type I interferons are well-known inducers of antiviral responses, in large part through the induction of transcription of interferon-inducible or -stimulated genes (ISGs). Given the critical role that APOBEC3 plays in controlling murine retrovirus infection, it is not surprising that the mouse and human *APOBEC3* genes are ISGs [22,35,36]. Although this aspect of APOBEC3 has not been explored in great detail in mice, it has been shown that interferon treatment of wild type but not A3 knockout mice and bone marrow-derived dendritic cells increases *Apobec3* transcription and renders them more resistant to MMTV infection [22]. This is interesting, given the observation that MMTV virions, which incorporate lipopolysaccharide in the viral membrane, activate Toll-like receptor (TLR) 4-dependent responses, including increasing type I interferon levels, during in vivo infection [37,38,39].

MLV has also been shown to induce type I interferons in mice, although via a different mechanism. TLRs recognize “pathogen-associated molecular patterns” (PAMPs) and belong to a class of anti-pathogen receptors that recognize different classes of PAMPs, including nucleic acids, proteins, and sugars; these are termed pattern recognition receptors (PRRs). PRRs ensure that host cells initiate a targeted response that ultimately rids the organism of harmful pathogens. Upon PAMP detection, PRRs initiate signaling cascades that induce expression of type I interferons and other cytokines and chemokines that increase the expression of ISGs and activate the adaptive immune system (see next section) [40,41,42]. In the case of MLV, the RNA-sensing TLR7, as well as several other nucleic acid sensing PRRs, absent in melanoma 2 (AIM2)-like receptors (ALRs), cyclic GMP-AMP synthase (cGAS), and members of the DEAD/H box (Asp-Glu-Ala-Asp/His) helicase, have all been implicated in the interferon response to MLV [23,43,44,45,46,47,48]. In particular, cGAS, ALR IFI203, and DDX41 are involved in the detection of incoming viral reverse transcripts in newly infected cells, leading to interferon production. Since APOBEC3 blocks reverse transcription, A3 knockout mice have a greater interferon response to MLV infection than do wild-type mice because there are more nucleic acid ligands produced that activate the sensing pathways [46]. The production of type I interferons through sensing likely “warns” bystander cells to arm themselves against infection, by increasing the levels of antiviral ISGs, particularly APOBEC3 [46]. Interestingly, injection of interferon diminished FV infection of wild-type but not A3 knockout mice, suggesting that APOBEC3 is the major anti-FV factor in mice [49].

## 5. Mouse APOBEC3 and the Virus-Neutralizing Antibody Response

As described above, the *Rfv3* allele in mice influences the antibody response to FV infection. The mechanism by which mAPOBEC3 promotes the neutralizing antibody response remains unclear. Several hypotheses have been proposed to explain this phenotype. APOBEC3 may promote the neutralizing antibody response by limiting FV-induced immune dysfunction. The suppression of virus infection by APOBEC3 limits the number of virus-producing cells in the early stages of infection. This may prevent FV-mediated damage of critical hematopoietic lineage cells required for generating an immune response [3,50]. Consistent with this idea, Δ5-expressing C57BL/6 wild-type mice have significantly lower FV-positive erythroid, myeloid, B, and T cells compared with A3 KO mice [51], suggesting that the protection of these cell subsets from FV infection may limit their immune dysfunction. The ability to produce CD8+ T cells as well as antibodies against sheep red blood cells is decreased in FV-infected mice, and both decreases were dependent on the loss of IFN-γ and increased IL-10 production [52]. Thus, FV-induced immune dysfunction may affect the neutralizing antibody response, and APOBEC3 prevents this immune dysfunction by limiting virus infection.

Maintenance of antibody responses in HIV-infected individuals is highly dependent on antigen levels [53,54,55]. Restriction factors inhibit viruses in infected cells and as a result, usually decrease antigen levels; thus, these factors are thought not to be optimal for driving B cell responses. Indeed, since APOBEC3 blocks reverse transcription, it likely reduces the production of viral immunogens. However, even in APOBEC3-expressing mice, there is some level of virus particle production, although the particles are less infectious [12,56]. These noninfectious particles are fusion-competent and could serve as immunogens to prime the B cell response [56].

APOBEC3 may also play a role in somatic hypermutation of immunoglobulin genes. Peripheral B cells can change their antibody affinity and isotype by somatically mutating their genomic DNA in response to infection. This mechanism of antibody diversity is tightly regulated and largely occurs through the action of another member of the cytidine deaminase family, AID (reviewed in [57]). Significantly lower levels of C-to-T and G-to-A somatic hypermutation and lower virus-binding ability were found in the Ig heavy-chain (IgH) sequences of FV-specific monoclonal antibodies derived from A3 knockout mice than those from wild-type mice [58]. This finding supports the idea that mAPOBEC3 plays a role in somatic hypermutation of immunoglobulin genes during antibody production, but whether the Ig somatic mutations are critical for the control of FV is not clear, since AID knockout mice, which are unable to undergo class-switch recombination, still control infection by producing virus-neutralizing IgM antibodies [59]. Moreover, since mAPOBEC3 is found in the cytoplasm, it is not clear when it has access to genomic DNA to mutate the *IgH* genes.

Restriction of FV infection and higher production of neutralizing antibodies depend on the relative amounts of the two splice isoforms of mAPOBEC3 [3]. However, it has recently been shown that the ability to produce neutralizing anti-FV antibodies depends more on interferon production than on the *Apobec3* allele [49]. Many stocks of FV contain an unrelated positive-strand, enveloped RNA nidovirus, lactate dehydrogenase virus (LDV) (reviewed in [60]). LDV affects both innate and adaptive immunity, and is a very potent stimulator of type I interferons via the TLR7 pathway [61]. The *Apobec3*-dependent generation of anti-FV antibodies only occurs in the presence of LDV because it depends on type I interferon signaling [49]. The effect of the *Apobec3* allele on type I interferon-mediated effects is likely due to the LDV-dependent induction of APOBEC3 RNA in mice expressing the +5 variant. It has been shown that in addition to higher basal levels of Δ5 compared to +5 APOBEC3, type I interferons induce even higher expression of the former than the latter. Thus, during FV infection, the more potent Δ5 protein likely limits virus levels and thereby blocks FV-mediated immune dysfunction caused by infection.

Given the recent finding that LDV-mediated production of type I interferon plays a critical role in the anti-FV antibody response, it is not surprising that neither MMTV or MMLV induce strong antibody responses in common inbred mouse strains, irrespective of the *Apobec3* allele [62]. Instead, the ability to produce antibodies to both viruses maps to the H2-Ob gene in the major histocompatibility locus [63]. This, along with the observation that in the absence of LDV virus, FV does not induce a strong antibody response, supports the findings that APOBEC3 restricts mouse retrovirus infection at an earlier step in the infection pathway e.g., by blocking reverse transcription.

## 6. Mouse Retroviruses Counteract Mouse APOBEC3

Retroviruses have evolved multiple means to counteract APOBEC3 proteins. One of them is exclusion of APOBEC3s from virions by virus-encoded proteins such as HIV’s virion infectivity factor (Vif), which causes the degradation of APOBEC3 proteins in virus producer cells [64,65]. However, while murine retroviruses package APOBEC3, they are not completely inhibited by it. The mode by which these viruses overcome restriction by mouse APOBEC3 is still not fully understood.

Virion-incorporated human APOBEC3 proteins induce hypermutation of the viral genome, which is the main contributor to the block in infection induced by these proteins [66]. Mouse APOBEC3, unlike human APOBEC3s, usually blocks infection by murine retroviruses without inducing or only inducing low-level G-to-A mutation [2,12,67]. In contrast, mouse APOBEC3 profoundly restricts Vif-deficient HIV-1, and the restriction includes high levels of G-to-A mutation [16,64,67]. Murine retroviruses have evolved specific ways to avoid being deaminated by mouse APOBEC3.

MMTV replication is suppressed by packaged mouse APOBEC3, while G-to-A mutations in the integrated proviral genome are rarely detected; instead, as discussed above, APOBEC3 blocks reverse transcription [12]. A recent study suggests that the alleviation of APOBEC3-mediated hypermutation by MMTV relies on its RT activity; MMTV may be resistant to mAPOBEC3 because its RT has a high processivity (DNA synthesis) rate compared to the HIV-1 RT, for example. Introduction of a mutation in the DNA polymerase domain of MMTV RT (F120L) that slowed processivity resulted in higher G-to-A mutation rates [68]. However, as discussed above, when human APOBEC3G transgenic mice were infected with MMTV, there was extensive deamination of the virus [19]. Moreover, the MMTV genome appears to have undergone APOBEC3-mediated selection for low GC content, thus making it somewhat resistant to cytidine deaminase-mediated mutations; this is similar to what has occurred with HIV/SIV and primate APOBEC3 proteins [69]. MMTV also uses a viral accessory protein called Rem to induce destruction of AID; this is thought to antagonize innate immunity during MMTV replication in lymphocytes [70]. Thus, it is possible that there are additional APOBEC3 antagonists encoded in the MMTV genome.

**Figure 2:**
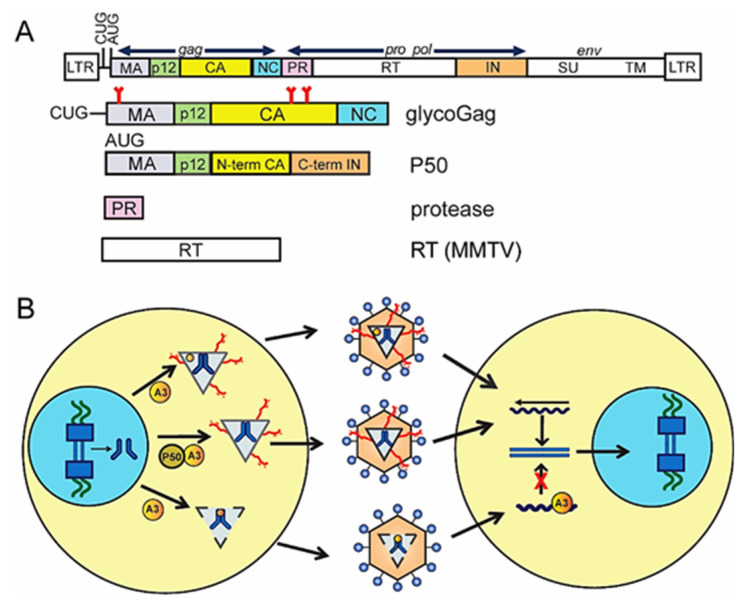
Mouse mammary tumor virus (MMTV) and murine leukemia virus (MLV) proteins that counteract APOBEC3. (**A**) Diagram of MLV genome and identified viral anti-APOBEC3 proteins. GlycoGag translation initiation from a CUG upstream from the Gag translation start site. Red lines indicate glycosylation sites. P50 is generated from an alternatively spliced mRNA that results in a protein encoding matrix (MA), p12, the N terminus of capsid (CA) and the C terminus of integrase (IN). The MLV PR has also been shown to degrade APOBEC3. Finally, it has been suggested that MMTV RT prevents APOBEC3 deamination activity by its rapid processivity. (**B**) The role of glycoGag and P50 in blocking APOBEC3. GlycoGag (red branched lines) prevents APOBEC3 from accessing the reverse transcription complex, and P50 prevents packaging of APOBEC3; both of proteins thereby facilitate reverse transcription in newly infected cells. Reverse transcription is inhibited by APOBEC3 after infection with virions lacking glycoGag, loss of which destabilizes the capsid, or lacking P50.

In contrast, there are known MLV-encoded proteins that counteract APOBEC3. Most gammaretroviruses, including the different MLVs, encode an alternate glycosylated form of the Gag polyprotein termed glycoGag, originating from an upstream CUG initiation codon that is in frame with the Gag polyprotein AUG translation start site [71,72,73] (Figure 2A). This results in the translation of a novel glycosylated polyprotein gPr80, which is proteolytically cleaved within to generate a 55 kD N-terminal membrane-associated peptide and a 40 kD C-terminal secreted peptide; both the 55 kD and gPr80 full-length proteins are found in virions [73,74,75,76]. A number of studies showed that glycoGag-mutant viruses were replication-competent in tissue culture cells but highly attenuated in vivo [77,78,79]. This difference between in vitro and in vivo infection was shown to be dependent on APOBEC3 [23,80]. Our lab found that the glycoGag-mutant viral particles are less stable than wild-type particles [23]. When glycoGag was absent, APOBEC3 blocked reverse transcription in cell culture and in vivo more effectively than it did with wild type virus [23,76]. glycoGag was also shown to inhibit the ability of mouse APOBEC3 to deaminate AKV MLV [81]. Capsid stability and both the deamination-dependent and -independent inhibitory activity of glycoGag were dependent, at least in part, on the glycosylation sites [76,81]. These studies combined suggest a mechanism in which glycoGag stabilizes the viral core and blocks APOBEC3 access to the reverse transcription complex, although exactly how this achieved is not clear (Figure 2B).

MLV is a simple retrovirus, and thus far, no Vif-like protein has been identified. However, viral proteins derived from alternatively spliced mRNAs are produced. Besides the full-length viral genomic and env RNAs, a 4.4-kb viral RNA transcript was identified in MLV infected cells [82]. This viral RNA, termed the alternative splice donor site (SD’) RNA, results from an alternative splice donor site in the gag region that uses the env splice acceptor site. The SD’ RNA encodes two viral proteins, P50 and P60 [83]. Our group found that P50, which contains MA, p12, the N terminus of CA and, the C terminus of IN, is a Vif-like protein that interacts with mouse APOBEC3 protein and blocks its packaging into virions through binding to CD2, the domain required for encapsidation [84] (Figure 2B). Unlike Vif, however, P50 does not cause proteolytic degradation of APOBEC3 but sequesters it from the assembling virus.

MLV may also use other means to counteract the inhibition by mouse APOBEC3. MLV viral RNA (vRNA) was reported to specifically block the mouse APOBEC3 incorporation into MLV virions by inhibiting its binding to Gag nucleocapsid protein [85]. In addition, the MLV PR may cleave packaged APOBEC3 after virion maturation and thereby may provide extra defense against APOBEC3 [85]. The cleavage site was found only in +5 but not Δ5 APOBEC3 (Figure 1A).

Thus, murine retroviruses have evolved multiple means to overcome the restriction by mouse APOBEC3, explaining their survival as infectious pathogens in mice. It is possible that additional APOBEC3-antagonizing measures in mouse retroviruses exist and await discovery.

## 7. Discussion

mAPOBEC3 plays an important role in modulating the pathogenicity of different mouse viruses, most of which cause cancer in mice and thereby shorten lifespan and reproductive capacity. APOBEC3 is probably one of the most critical factors that keep infection levels low, thereby allowing both survival of the infected organism and transmission of the virus to offspring. The primary mechanism for inhibition is cytosine deamination-independent inhibition of reverse transcription rather than cytidine deamination. What is yet to be determined is mechanistically how mAPOBEC3 is prevented from deaminating murine retroviral genomes.

The different murine retroviruses have responded with a variety of strategies to avoid APOBEC3-mediated restriction, including the glycosylated form of Gag, the P50 proteins encoded in MLV, and the highly processive RT encoded by MMTV. More studies are needed to determine how these proteins function mechanistically. It is also possible that more mAPOBEC3 antagonizing mouse retrovirus-encoded factors exist and are yet to be discovered and studied.

In summary, mouse models have provided an important system to study viruses in their natural hosts, allowing us to understand APOBEC3 protein in vivo action and the co-evolution of viruses and their hosts.
